# In Situ Construction of Cu^+^‐O_v_‐Ce^3+^ Sites on CeO_2_ for Efficient NH_3_ Oxidation

**DOI:** 10.1002/advs.202511023

**Published:** 2025-09-18

**Authors:** Chaomin Duan, Meng Wang, Yan Zhang, Zhihua Lian, Wenpo Shan

**Affiliations:** ^1^ State Key Laboratory of Advanced Environmental Technology, Institute of Urban Environment Chinese Academy of Sciences Xiamen 361021 China; ^2^ Zhejiang Key Laboratory of Pollution Control for Port‐Petrochemical Industry Ningbo Urban Environment Observation and Research Station Institute of Urban Environment Chinese Academy of Sciences Ningbo 315800 China; ^3^ University of Chinese Academy of Sciences Beijing 100049 China

**Keywords:** bidentate nitrate, CeCuO_*x*_ catalyst, Cu^+^‐O_v_‐Ce^3+^ site, monodentate nitrate, NH_3_ oxidation

## Abstract

Ammonia (NH_3_) emissions adversely affect both the environment and human health. The selective catalytic oxidation of NH_3_ (NH_3_‐SCO) holds great promise for NH_3_ abatement; however, there remains a lack of cost‐effective NH_3_‐SCO catalysts with high activity and N_2_ selectivity for practical applications. This study reports on a strategy for constructing an outstanding NH_3_ oxidation catalyst via the in situ doping of Cu into CeO_2_ nanorods. The designed CeCuO*_x_* nanorod catalyst shows remarkable activity, N_2_ selectivity, and stability for NH_3_ oxidation, achieving complete conversion below 250 °C, with ≈80% N_2_ selectivity. The results of various characterization tests and density functional theory (DFT) calculations show that the addition of Cu induces the formation of abundant Cu^+^‐O_v_‐Ce^3+^ active sites on the CeO_2_, which are beneficial for the adsorption and activation of reactants. Additionally, in situ diffuse reflectance infrared Fourier transform spectroscopy (DRIFTS) confirms that the addition of Cu switches the primary intermediate species of NH_3_ oxidation from bidentate nitrate species to monodentate nitrate species, which accelerates the rate‐determining step of NH_3_‐to‐N_2_ oxidation. Therefore, doping Cu into CeO_2_ greatly improves its NH_3_ oxidation activity and N_2_ selectivity. This study provides valuable insights into the construction of highly active sites for NH_3_ oxidation.

## Graphical abstract

An advanced CeCuO*_x_* nanorod catalyst is developed via in situ hydrothermal synthesis, and it exhibits remarkable NH_3_‐SCO activity, N_2_ selectivity, and stability. The addition of Cu induces the formation of abundant Cu^+^‐O_v_‐Ce^3+^ active sites on CeO_2_ and switch the primary intermediate species of NH_3_ oxidation from bidentate nitrate species to monodentate nitrate species.

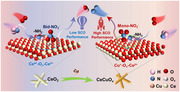

## 1 Introduction

Ammonia (NH_3_) emissions from stationary and mobile sources not only severely harm human health but also significantly contribute to fine particulate matter (PM_2.5_) formation, deteriorating the atmospheric environment, which is of note considering the planned application of NH_3_ as a fuel in the near future.^[^ ^1^, ^2^, ^3^, ^4^ ^]^ Various technologies have been developed to eliminate NH_3_, including adsorption,^[^ ^5^ ^]^ biofiltration,^[^ ^6^ ^]^ catalytic combustion,^[^ ^7^ ^]^ catalytic oxidation,^[^ ^8^ ^]^ chemical treatment,^[^ ^9^ ^]^ and photocatalytic oxidation.^[^ ^10^ ^]^ The selective catalytic oxidation of NH_3_ to N_2_ and H_2_O (NH_3_‐SCO) is the most effective technology for NH_3_ removal, and the key to this process lies in the development of high‐efficiency NH_3_‐SCO catalysts.^[^ ^11^ ^]^

To date, various catalysts have been developed, including noble metal,^[^ ^12^ ^]^ non‐noble metal,^[^ ^13^ ^]^ and zeolite catalysts.^[^ ^14^ ^]^ Notably, supported noble‐metal Pt/Pd‐based catalysts are the primary commercial catalysts for NH_3_‐SCO owing to their superior NH_3_ oxidation performance. However, the scarcity and high cost of noble metals create an urgent need for the development of cost‐effective non‐noble‐metal catalysts with high efficiency and superior N_2_ selectivity for NH_3_‐SCO.^[^ ^15^ ^]^ Cerium dioxide (CeO_2_) has attracted considerable attention for use in catalysis owing to its excellent oxygen storage and release abilities.^[^ ^16^ ^]^ However, pure CeO_2_ exhibits poor NH_3_‐SCO activity, with complete NH_3_ conversion at 420 °C and poor N_2_ selectivity.^[^ ^17^ ^]^ Many strategies have been explored to further improve the NH_3_ oxidation activity of CeO_2_. Among them, doping with transition metals, especially Cu, is attracting growing interest in catalytic reactions because of the low cost and excellent redox capabilities of transition metals.^[^ ^18^, ^19^ ^]^ For example, Cheng et al.^[^ ^20^ ^]^ prepared a series of CeO_2_‐based catalysts by doping Co, Fe, Cu, and Zr into CeO_2_ and revealed that Cu/CeO_2_ showed optimal performance, combining high NH_3_ conversion with favorable N_2_ selectivity. Wang et al.^[^ ^21^ ^]^ synthesized a CuO‐CeO_2_ mixed oxide by using the surfactant‐templated method, which achieved complete NH_3_ conversion at ≈250 °C. Sun et al.^[^ ^22^ ^]^ supported Cu on CeO_2_ with different shapes (nanorods and nanocubes) and found that Cu/Ce‐NR with a nanorod morphology possessed higher catalytic activity (T_100_ = 240 °C) for NH_3_‐SCO than that of Cu/Ce‐NC with a nanocube morphology (T_100_ = 270 °C). In summary, the introduction of Cu into CeO_2_ catalysts offers an effective strategy for enhancing NH_3_‐SCO activity and N_2_ selectivity. However, research on copper‐ceria catalysts has primarily focused on the effects of synthesis methods or the CeO_2_ morphology. The formation mechanism of active sites in highly active copper‐ceria catalysts, as well as the influence of these active sites on the reaction pathways of the NH_3_‐SCO reaction, requires further exploration.

This study used in situ hydrothermal synthesis to develop an excellent CeCuO*_x_* nanorod catalyst, which exhibited superior NH_3_‐SCO activity, N_2_ selectivity, and stability. Various characterization techniques and density functional theory (DFT) calculations were employed to explore the role of Cu doping into CeO_2_ in enhancing the catalytic activity of CeO_2_ and to reveal the relationship between the structural properties of the catalysts and their catalytic performance. Additionally, the NH_3_ oxidation reaction mechanisms of CeO_2_ catalysts with and without Cu doping were clearly elucidated. The results enrich the understanding of the active sites and reaction mechanism for NH_3_‐SCO and will aid in the development of high‐efficiency catalysts for the NH_3_‐SCO reaction.

## 2 Results and Discussion

### 2.1 Catalytic Activities, Stability, and Kinetic Studies

**Figure 1:**
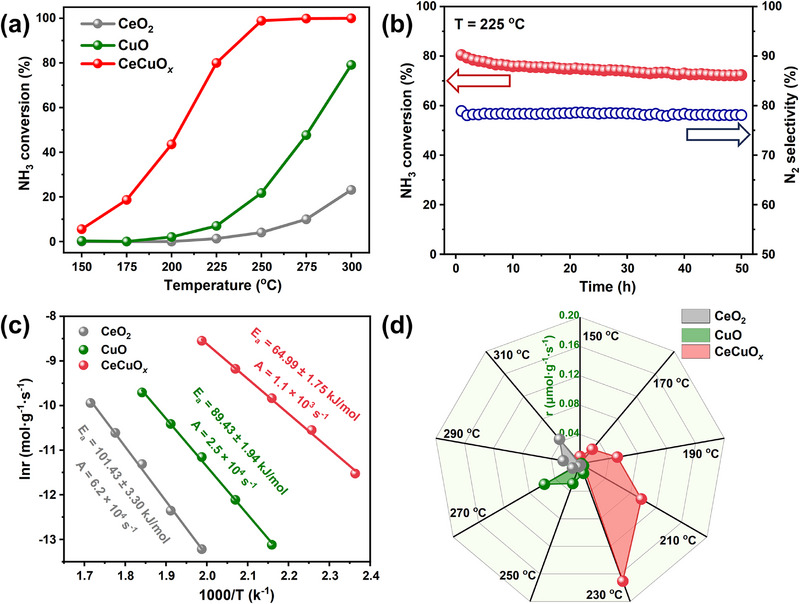
a) NH_3_ conversion of as‐prepared catalysts. b) NH_3_ conversion and N_2_ selectivity of the CeCuO*_x_* catalyst during a long‐term stability test at 225 °C. Reaction conditions: 500 ppm of NH_3_ and 10 vol.% O_2_ in N_2_ balance, and weight hourly space velocity (WHSV) = 120 000 mL g^−1^ h^−1^. c) Arrhenius plots and d) reaction rate of the NH_3_ oxidation for the as‐prepared catalysts. Reaction conditions: 500 ppm of NH_3_ and 10 vol.% O_2_ in N_2_ balance, WHSV of CeO_2_ = 400 000 mL g^−1^ h^−1^, WHSV of CuO = 600 000 mL g^−1^ h^−1^, and WHSV of CeCuO*_x_* = 1 200 000 mL ^−1^g h^−1^.

As shown in **Figures** **1**a and S1a,b (Supporting Information), the pure CeO_2_ and CuO catalysts showed poor NH_3_‐SCO activity, with NH_3_ conversion remaining below 80% and N_2_ selectivity of 70% at 300 °C. Surprisingly, for NH_3_ oxidation, the catalytic performance of the CeCuO*_x_* catalyst was significantly enhanced by the addition of Cu to CeO_2_, achieving 100% conversion of NH_3_ at 250 °C while simultaneously increasing N_2_ selectivity to ≈80% (Figure S1c, Supporting Information). In this study, the performances of CeCuO*_x_* catalysts with different Cu contents were investigated to explore their dependence on the Cu content. As shown in Figure S2 (Supporting Information), the CeCuO*_x_* catalyst with a higher Cu content exhibited higher NH_3_ oxidation activity than that with a low Cu content, and the optimized Ce/Cu ratio was 9/1. In addition, under practical application conditions, the long‐term stability of NH_3_‐SCO catalysts is crucial. As shown in Figure S3 (Supporting Information), the catalytic activity and N_2_ selectivity of the CeO_2_ catalyst exhibited a continuous declining trend during the 50‐h stability test. Similarly, the NH_3_ oxidation activity of the CuO catalyst decreased during the 50‐h stability test, while the N_2_ selectivity was stable throughout the evaluation period. Figure 1b shows that the NH_3_ oxidation activity of the CeCuO*_x_* catalyst decreased slightly, and the N_2_ selectivity remained unchanged during the 50‐h test. This indicated that a cheap, effective, and stable catalyst for NH_3_ oxidation was developed.

Kinetic studies were also performed to further analyze the effect of Cu doping on the intrinsic activity of the CeCuO*_x_* catalyst. As shown in Figure 1c, the activation energy (*E_a_*) values of the CeO_2_ and CuO catalysts were 101.43 ± 3.30 and 89.43 ± 1.94 kJ mol^−1^, respectively. Following the doping of Cu into CeO_2_, the *E_a_* greatly decreased to 64.99 ± 1.75 kJ mol^−1^, indicating that Cu doping had a positive effect on decreasing the reaction barrier. Furthermore, the addition of Cu could also be beneficial to improving the reaction rate (Figure 1d).

### 2.2 Morphological and Structural Characteristics

**Figure 2:**
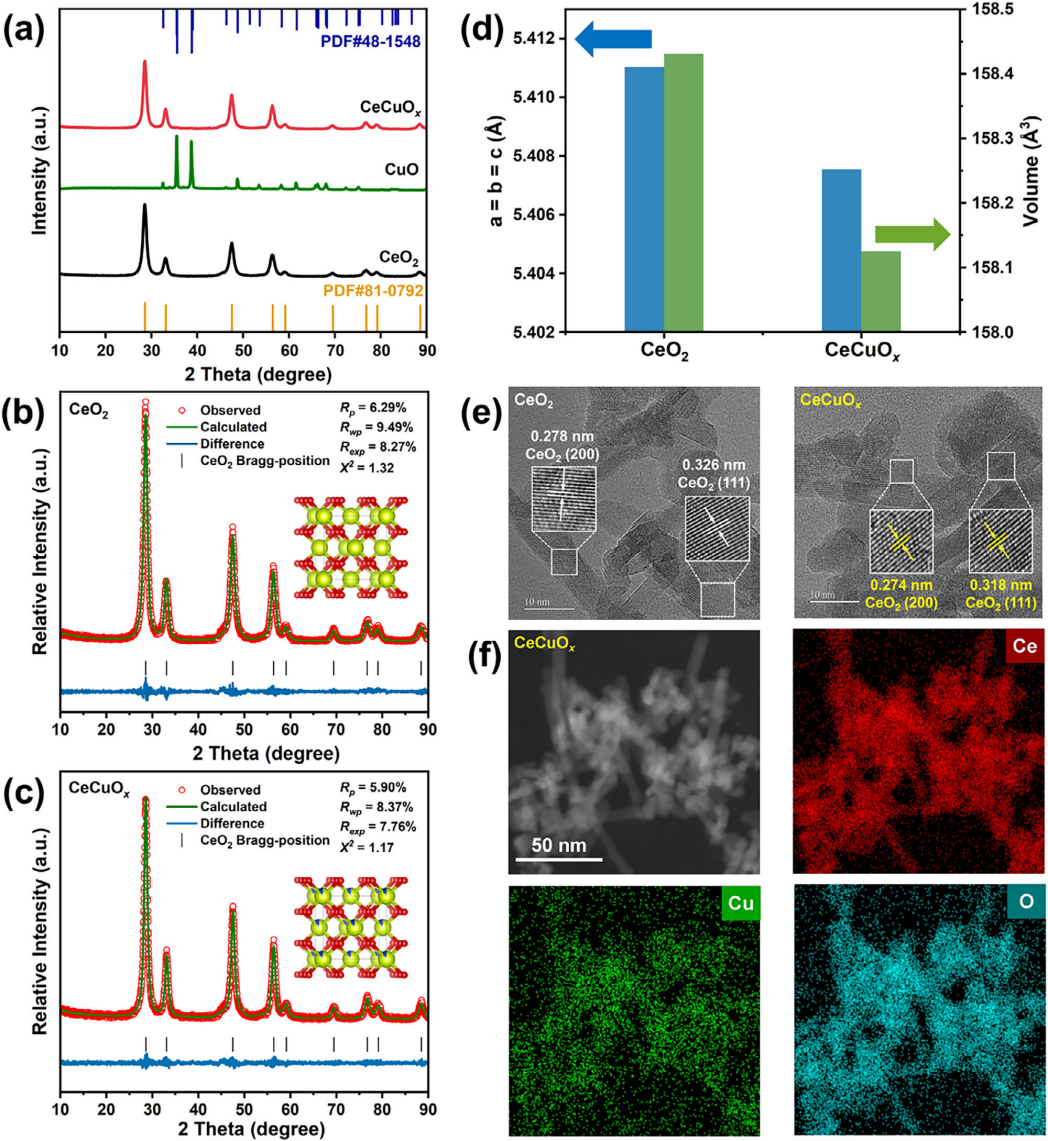
a) XRD patterns of as‐prepared catalysts. Rietveld‐refined XRD patterns of b) CeO_2_ and c) CeCuO*_x_* catalysts. d) Rietveld‐refined parameters of CeO_2_ and CeCuO*_x_* catalysts. e) HRTEM images of CeO_2_ and CeCuO*_x_* catalysts. f) STEM‐EDS mapping images of the CeCuO*_x_* catalyst.

**Figure** **2**a shows the X‐ray diffraction (XRD) patterns of the as‐prepared catalysts. The CeO_2_ and CeCuO*_x_* catalysts exhibited the typical diffraction pattern of CeO_2_ with a cubic fluorite structure (JCPDS 81‐0792). Meanwhile, the pure CuO catalyst showed a monoclinic structure of CuO crystals (JCPDS 48‐1548) with no other impurities observed. No diffraction peaks of Cu species were observed in the pattern of the CeCuO*_x_* catalyst, which could be due to Cu being doped into CeO_2_, and/or being well‐dispersed on the surface of CeO_2_.^[^ ^23^ ^]^ To confirm the incorporation of Cu into the CeO_2_ lattice, XRD refinement was performed; the results are shown in Figure 2b–d. As shown in Figure 2d, the lattice constants of pure CeO_2_ were a = b = c = 5.411 Å, while with the doping of Cu, the lattice constants notably decreased, indicating that some of the Ce atoms were substituted by Cu atoms with a smaller radius. To evaluate the stability of the post‐reaction CeCuO*_x_* catalyst, XRD characterization was performed. Figure S4 (Supporting Information) clearly shows that, after the catalytic test, the CeCuO*_x_* catalyst exhibited almost the same diffraction peaks as the fresh catalyst, indicating that the CeCuO*_x_* catalyst was stable for NH_3_ oxidation.

As shown in Figure S5a (Supporting Information), both the CeO_2_ and CeCuO*_x_* catalysts exhibited type‐IV adsorption isotherms with H3‐type hysteresis loops, indicating that the pore structures of the CeO_2_ and CeCuO*_x_* catalysts were mesoporous.^[^ ^24^ ^]^ Figure S5b (Supporting Information) shows the pore size distributions of the CeO_2_ and CeCuO*_x_* catalysts. The pore sizes of both catalysts were predominantly concentrated in the range of 2–3 nm. The Brunauer‐Emmet‐Teller (BET) surface areas, pore volumes, and average pore sizes of the catalysts are presented in Table S1 (Supporting Information). As shown in Table S1 (Supporting Information), with the addition of Cu, the BET surface area, pore volume, and average pore size of the catalyst decreased slightly, which could be related to the blockage of some of the interparticle mesopores by doped Cu species during calcination.

The morphologies of the catalysts were investigated using transmission electron microscopy (TEM), and the corresponding images are shown in Figures 2e,f and S6 (Supporting Information). As shown in Figure S6 (Supporting Information), the catalysts with and without Cu doping exhibited a nanorod morphology, indicating that Cu doping did not affect the morphology of the catalysts. Figure 2e shows that both the CeO_2_ and CeCuO*_x_* catalysts showed well‐defined lattice fringes. The widths of the lattice fringes were 0.278 and 0.326 nm for pure CeO_2_, corresponding to the diffraction patterns of the (200) and (111) zone axes of CeO_2_, respectively. Interestingly, with the addition of Cu, the width of the lattice fringes decreased, which was attributed to the incorporation of Cu into CeO_2_ during calcination, leading to lattice shrinkage. Although both the (200) and (111) planes were exposed in the CeO_2_ and CeCuO*_x_* catalysts, the (111) plane was the predominantly exposed surface, as shown in Figure S7 (Supporting Information). In addition, the elemental distributions of the catalysts were investigated using scanning transmission electron microscopy‐energy‐dispersive X‐ray spectroscopy (STEM‐EDS) mapping. Figures 2f and S8 (Supporting Information) show that Cu was well‐dispersed in the CeCuO*_x_* nanorods, consistent with the XRD results.

### 2.3 Redox Properties

**Figure 3:**
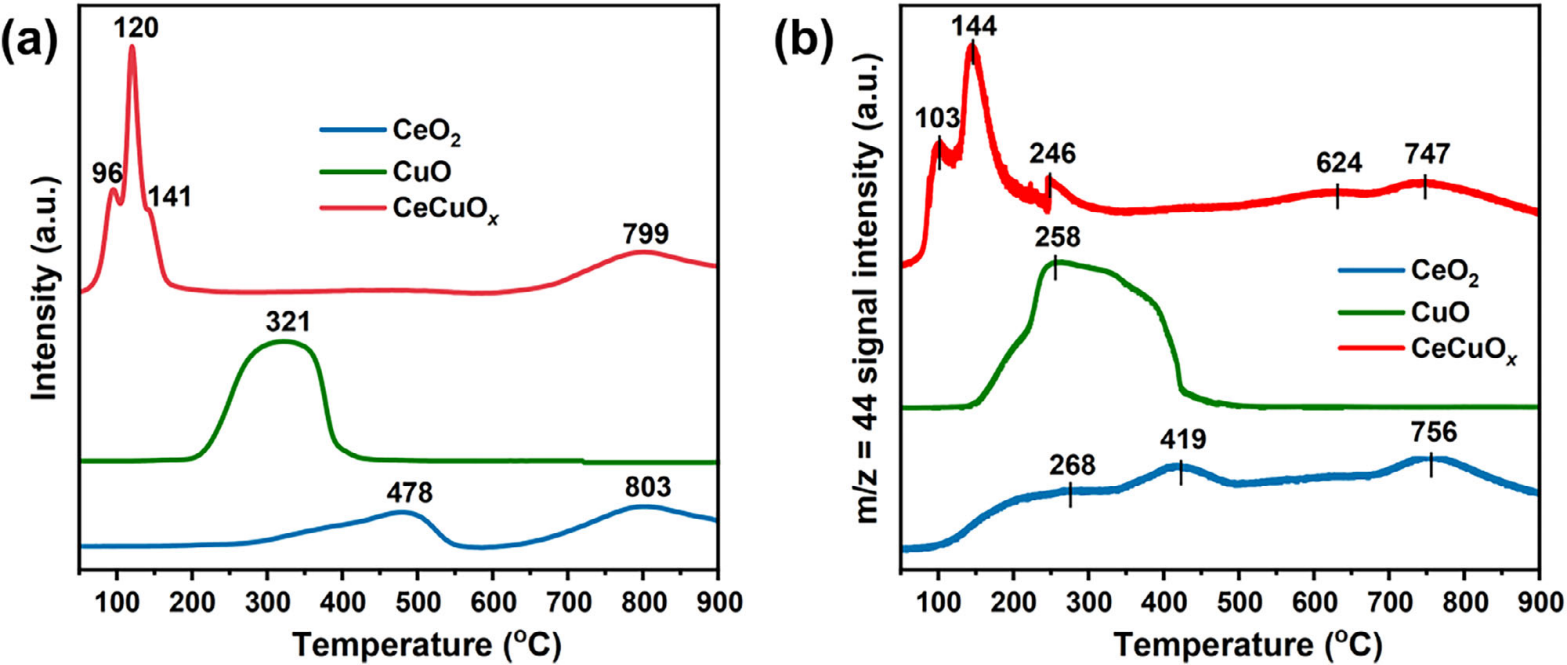
a) H_2_‐TPR and b) CO‐TPR profiles of the as‐prepared catalysts.

The state of the Cu species and redox properties of the as‐prepared catalysts were determined through H_2_/CO temperature‐programmed reduction (H_2_‐TPR, CO‐TPR) experiments. As shown in **Figure** **3**a, the H_2_ reduction profile of pure CeO_2_ exhibited two distinct peaks, located at 478 and 803 °C, which could be assigned to the reduction of surface Ce^4+^ and bulk Ce^4+^, respectively.^[^ ^25^ ^]^ The pure CuO catalyst exhibited a reduction peak at 321 °C, corresponding to bulk CuO*_x_*.^[^ ^22^ ^]^ With the addition of Cu to CeO_2_, the CeCuO*_x_* catalyst displayed four reduction peaks. The consumption peak centered at 96 °C was ascribed to the reduction of well‐dispersed CuO*_x_* clusters.^[^ ^26^, ^27^ ^]^ The sharp peak at 120 °C is attributed to the reduction of Cu─O─Ce species,^[^ ^28^, ^29^, ^30^ ^]^ and the peak at 141 °C was associated with the reduction of bulk CuO*_x_* species.^[^ ^31^, ^32^ ^]^

To further explore the interaction between Cu and CeO_2_, CO‐TPR experiments were also performed. For the CeO_2_ catalyst, a weak CO_2_ signal was detected at ≈268 °C, which was related to the reduction of the surface‐adsorbed oxygen species of CeO_2_ (Figure 3b). The peak located at 419 °C corresponded to CO oxidation via a reaction with surface lattice oxygen species, and the peak at 756 °C was associated with the bulk lattice oxygen species of CeO_2_. Pure CuO displayed a broad and asymmetric CO_2_ desorption peak at 150–450 °C, attributed to the reduction of bulk CuO*_x_*.^[^ ^33^ ^]^ With the doping of Cu into CeO_2_, two new CO_2_ peaks were detected in the range of 50–200 °C. Based on the H_2_‐TPR results, these peaks were attributed to the reduction of well‐dispersed CuO*_x_* clusters and Cu─O─Ce species, respectively. Meanwhile, the peak at 246 °C could be assigned to either bulk CuO*_x_* or surface‐adsorbed oxygen species. The peak at 624 °C was ascribed to the reaction of CO with surrounding lattice oxygen from Cu‐doped CeO_2_.^[^ ^24^ ^]^ These results indicate that Cu was embedded into the CeO_2_ lattice and induced the formation of Cu─O─Ce species.

### 2.4 Analysis of O_v_ and Construction of Cu^+^‐O_v_‐Ce^3+^

**Figure 4:**
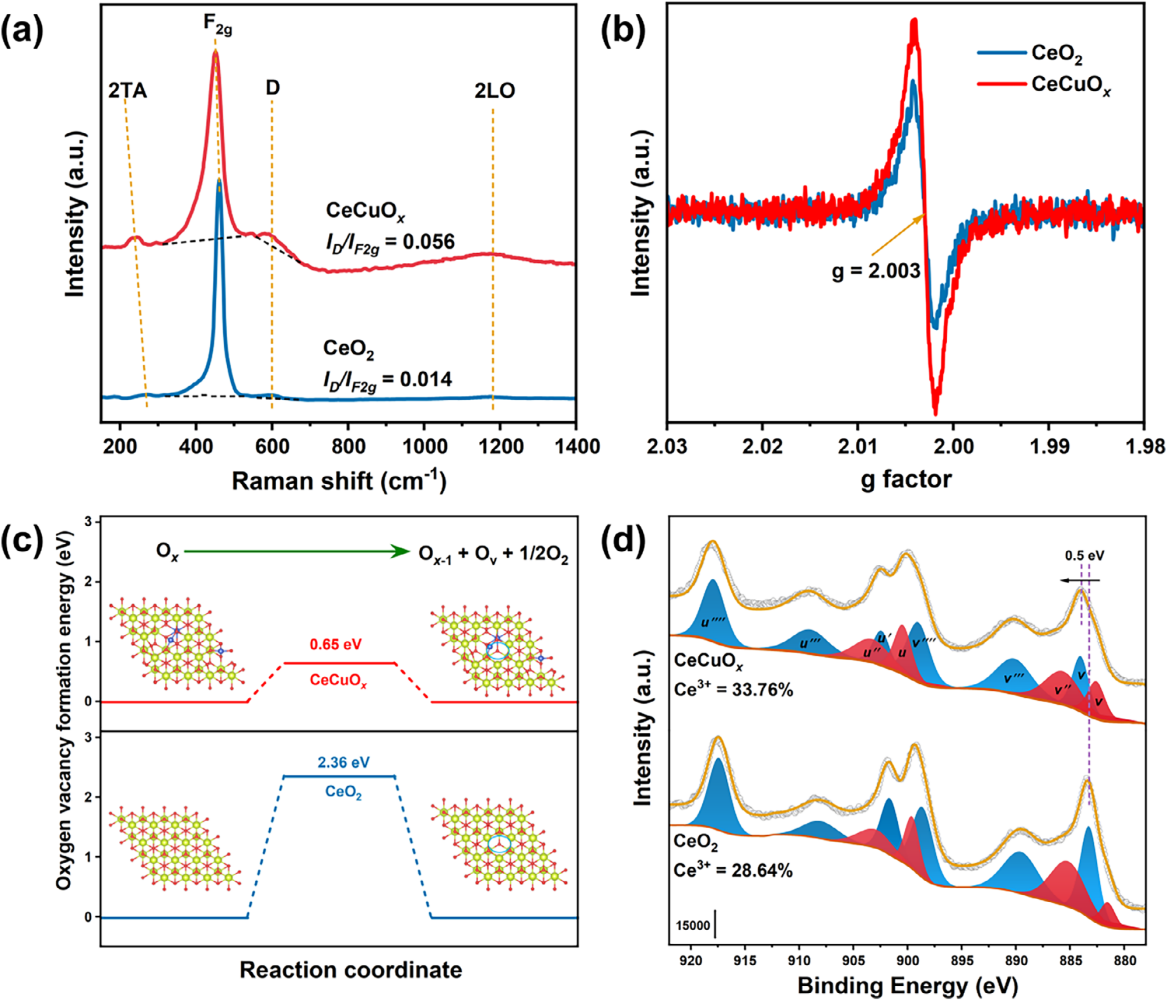
a) Raman spectra, b) EPR spectra, c) oxygen‐vacancy formation energy (light green, Ce atoms; red, O atoms; blue, Cu atoms), and d) Ce 3d XPS results of the CeO_2_ and CeCuO*_x_* catalysts.

Raman and X‐band electron paramagnetic resonance (EPR) tests were performed to explore the effect of Cu doping on the generation of oxygen vacancies (O_v_). As shown in **Figure** **4**a, the Raman spectrum of the CeO_2_ catalyst exhibited four peaks, located at 272, 461, 590, and 1170 cm^−1^. The peaks at 280 and 1170 cm^−1^ were attributed to the second‐order transverse acoustic (2TA) and second‐order longitudinal optical (2LO) vibration modes of fluorite CeO_2_, respectively. The prominent peaks at 461 and 590 cm^−1^ were assigned to the lattice vibration mode (F_2g_) and the defect‐induced (D) mode of fluorite CeO_2_, respectively.^[^ ^24^, ^34^ ^]^ With the addition of Cu, the CeCuO*_x_* catalyst also showed four vibration peaks. However, compared with the CeO_2_ catalyst, the 2TA and F_2g_ bands of the CeCuO*_x_* catalyst exhibited a distinct redshift, attributed to the extension of the Ce─O bond length, and the formation of Cu─O─Ce species was induced.^[^ ^35^ ^]^ The ratio of the integral areas for the D peak to those for the F_2g_ peak (*I_D_/I_F2g_*) was related to the relative amount of O_v_ on the surface of the catalysts.^[^ ^36^ ^]^ The CeCuO*_x_* catalyst exhibited a significantly higher *I_D_/I_F2g_* value than that of the CeO_2_ catalyst, which indicated that doping with Cu was beneficial for the formation of O_v_. In addition, the EPR results confirmed that the incorporation of Cu promoted the generation of O_v_ in the catalyst (Figure 4b).^[^ ^37^ ^]^ To further explore the effect of Cu doping on the formation of O_v_, the formation energy of O_v_ was calculated. The CeCuO*_x_* catalyst exhibited a lower O_v_ formation energy (1.23 eV) than that of CeO_2_ (2.35 eV) (Figure 4c). Therefore, the in situ doping of Cu into CeO_2_ was beneficial for the formation of O_v_.

The effect of Cu doping on the surface chemical valence states of CeO_2_ was studied using X‐ray photoelectron spectroscopy (XPS). As shown in Figure 4d, the Ce 3d XPS spectrum of the CeO_2_ catalyst could be fitted to ten peaks. The peaks labeled v, v“”, u, and u“” could be attributed to Ce^3+^, while the other peaks were ascribed to Ce^4+^.^[^ ^38^ ^]^ The ratios of Ce^3+^/(Ce^3+^ + Ce^4+^) for the CeO_2_ and CeCuO*_x_* catalysts were calculated. With the addition of Cu, the ratio of Ce^3+^/(Ce^3+^ + Ce^4+^) increased greatly (from 28.64% to 33.76%), suggesting that Cu doping induced the formation of more O_v_‐Ce^3+^ on the surface of CeO_2_.^[^ ^39^ ^]^ In addition, the peaks shifted toward higher binding energies after the addition of Cu, which was attributed to the partial reduction of Ce^4+^ to Ce^3+^ due to the formation of oxygen vacancies and electron transfer from Ce to Cu. These observations are consistent with the increased Ce^3+^ content and elevated oxygen‐vacancy concentration.^[^ ^21^, ^40^ ^]^ The charge density differences of the CeO_2_ and CeCuO*_x_* catalysts (Figure S9, Supporting Information) also confirmed that electron transfer occurred during the formation of O_v_, with electrons mainly accumulating around the O_v_. Figure S10a (Supporting Information) shows the Cu 2p XPS spectrum, which could be deconvoluted into seven peaks. The v and u peaks corresponded to Cu^+^, the v' and u' peaks were assigned to Cu^2+^, and the v“”, v“”', and v“”“” peaks were shake‐up satellite peaks.^[^ ^22^ ^]^ The area ratio of the satellite peaks to that of the main peaks (*I_sat_/I_mp_*) was used to analyze the valence of the Cu species. The *I_sat_/I_mp_* ratio of the CeCuO*_x_* catalyst was 0.32, which is lower than the typical value for pure CuO (0.57).^[^ ^41^ ^]^ This suggests that copious amounts of Cu^+^ species were present in the CeCuO*_x_* catalysts. The valence distribution of Cu for the CeCuO*_x_* catalyst was further investigated using Cu LMM Auger spectroscopy (Figure S10b, Supporting Information). The peaks at ≈570.5 and 577.1 eV corresponded to Cu^+^ and Cu^2+^ species, respectively. The ratio of Cu^+^/(Cu^+^ + Cu^2+^) was 77.01%, indicating that the CeCuO*_x_* catalyst contained abundant Cu^+^ species. UV–vis was obtained to further assess the electronic structures and surface defects. Both the CeO_2_ and CeCuO*_x_* catalysts exhibited three characteristic peaks centered at 231, 265, and 352 nm (Figure S11a, Supporting Information), which could be attributed to the f → d band transition of Ce^3+^ species, charge transfer from low‐coordinated Ce^4+^ to O, and interband transitions, respectively.^[^ ^42^ ^]^ The shoulder peak at 483 nm for the CeCuO*_x_* catalyst was ascribed to the emission and absorption of a single photon by Cu^+^ complexes.^[^ ^43^ ^]^ Additionally, the peak located at 674 nm was attributed to the d‐band transitions of Cu^2+^ in an octahedral O_h_ configuration, showing a tetragonal distortion. The transition from O^2−^ to Ce^3+^ was associated with the defects in the catalyst, and the increased Ce^3+^ content with Cu doping led to a narrowing of the band gap (*E_g_*) (Figure S11b, Supporting Information).^[^ ^44^ ^]^ Interestingly, Cu doping into CeO_2_ also led to an increase in the ratio of Ce^3+^. This reflected the fact that, in the interaction of Cu with CeO_2_, electrons were transferred from Ce─O to Cu─O, leading to the weakening of the Ce─O bond. The doped Cu species had a lower valence than that of Ce, which facilitated the removal of lattice oxygen in Cu^2+^‐O_2_‐Ce^4+^ via charge compensation, promoting the generation of O_v_. Moreover, the formation of O_v_ may be accompanied by a decrease in the valence states of Cu^2+^ and Ce^4+^ to maintain the charge balance, resulting in the formation of Cu^+^‐O_v_‐Ce^3+^.^[^ ^45^ ^]^

### 2.5 Oxygen Activation Performance

**Figure 5:**
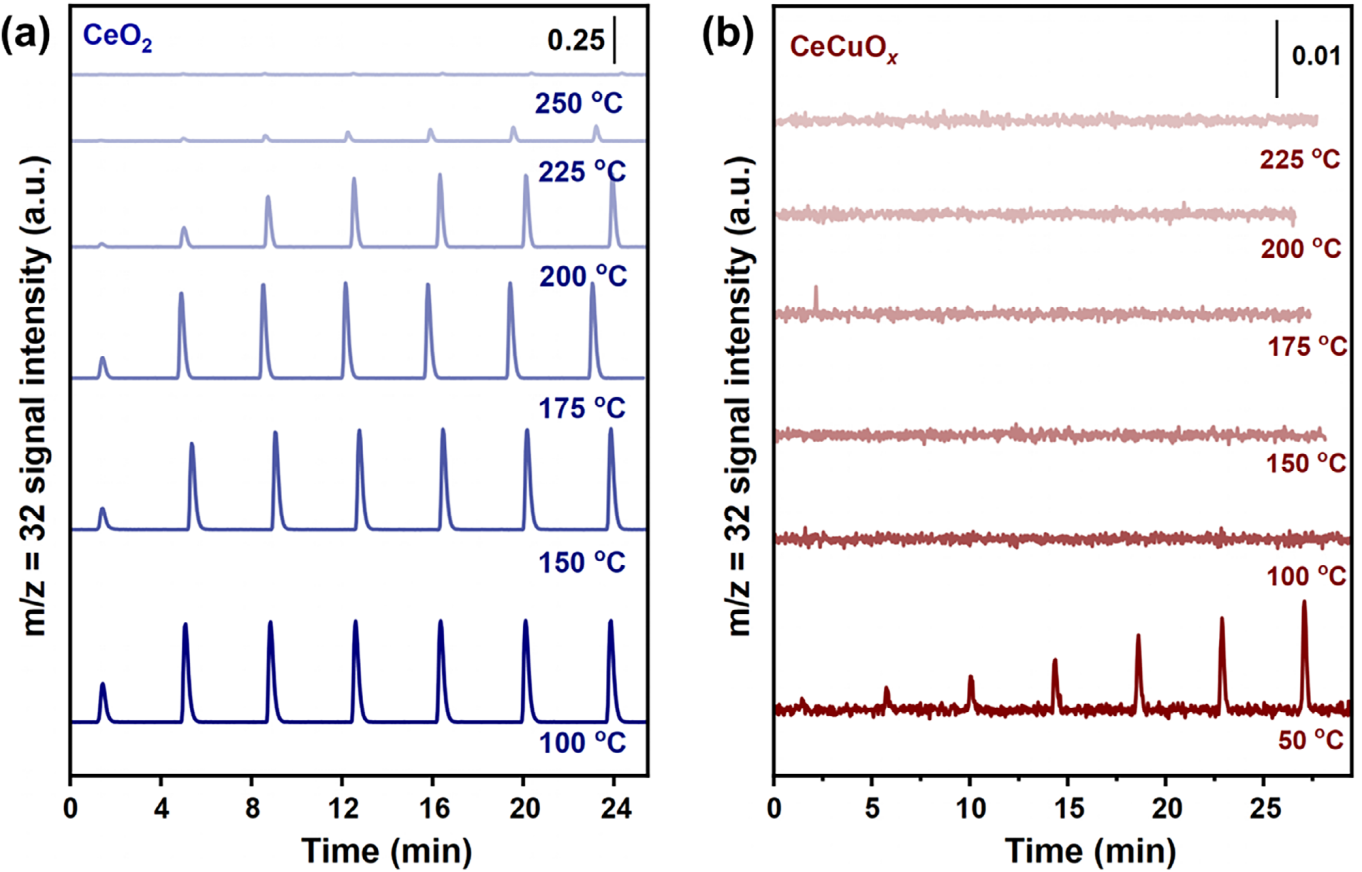
In situ O_2_‐pulse spectra of O_2_ signals for a) CeO_2_ and b) CeCuO*_x_* catalysts.

In situ O_2_‐pulse, O_2_ temperature‐programmed desorption (O_2_‐TPD), and O 1s XPS were used to assess the oxygen activation ability of the CeO_2_ and CeCuO*_x_* catalysts, and the results are shown in **Figure** **5**, Figures S12 and S13 (Supporting Information), respectively. As shown in Figures 5a and S12a (Supporting Information), for the pure CeO_2_ catalyst, the injected oxygen was completely activated at 250 °C. With the doping of Cu into the catalyst, the temperature of oxygen activation decreased from 250 to 100 °C, indicating that the Cu doping was beneficial for the activation of gaseous oxygen and promoted the generation of more active oxygen species (Figure 5b; Figure S12b, Supporting Information). The O_2_‐TPD and O 1s XPS results also confirmed that the addition of Cu promoted the activation of oxygen (Figure S13, Supporting Information). Combined with the results of Raman spectroscopy, Ce 3d XPS, O 1s XPS, EPR, O_2_‐TPD, in situ O_2_−pulse, and DFT, it can be confirmed that Cu doping into CeO_2_ induced the formation of abundant Cu^+^‐O_v_‐Ce^3+^ sites, which was beneficial to promoting the adsorption and activation of oxygen.

### 2.6 Reaction Mechanism Analysis

NH_3_ adsorption is a critical step in the NH_3_ oxidation reaction, and acidic sites are closely associated with the ability to adsorb and activate NH_3_.^[^ ^46^, ^47^ ^]^ To explore the effect of Cu doping on the acidic sites of CeO_2_, NH_3_ temperature‐programmed desorption (NH_3_‐TPD) was applied. The NH_3_ desorption curves of the CeO_2_ and CeCuO*_x_* catalysts could be divided into three NH_3_ desorption peaks, corresponding to weak acidic sites (≈97 °C), medium acidic sites (≈144 °C), and strong acidic sites (≈200 °C), respectively (Figure S14a, Supporting Information).^[^ ^48^ ^]^ The acidic sites on the catalysts were quantified by calculating the integral areas of the NH_3_‐TPD curves. As shown in Figure S14b (Supporting Information), with the addition of Cu, the ratio of the peak areas of different acid sites to the BET surface areas increased markedly, indicating that Cu doping facilitated NH_3_ adsorption. In addition, the NH_3_ adsorption energy was calculated. Figure S14c (Supporting Information) shows that the addition of Cu had a positive effect on the adsorption of NH_3_, which is consistent with the NH_3_‐TPD results.

**Figure 6:**
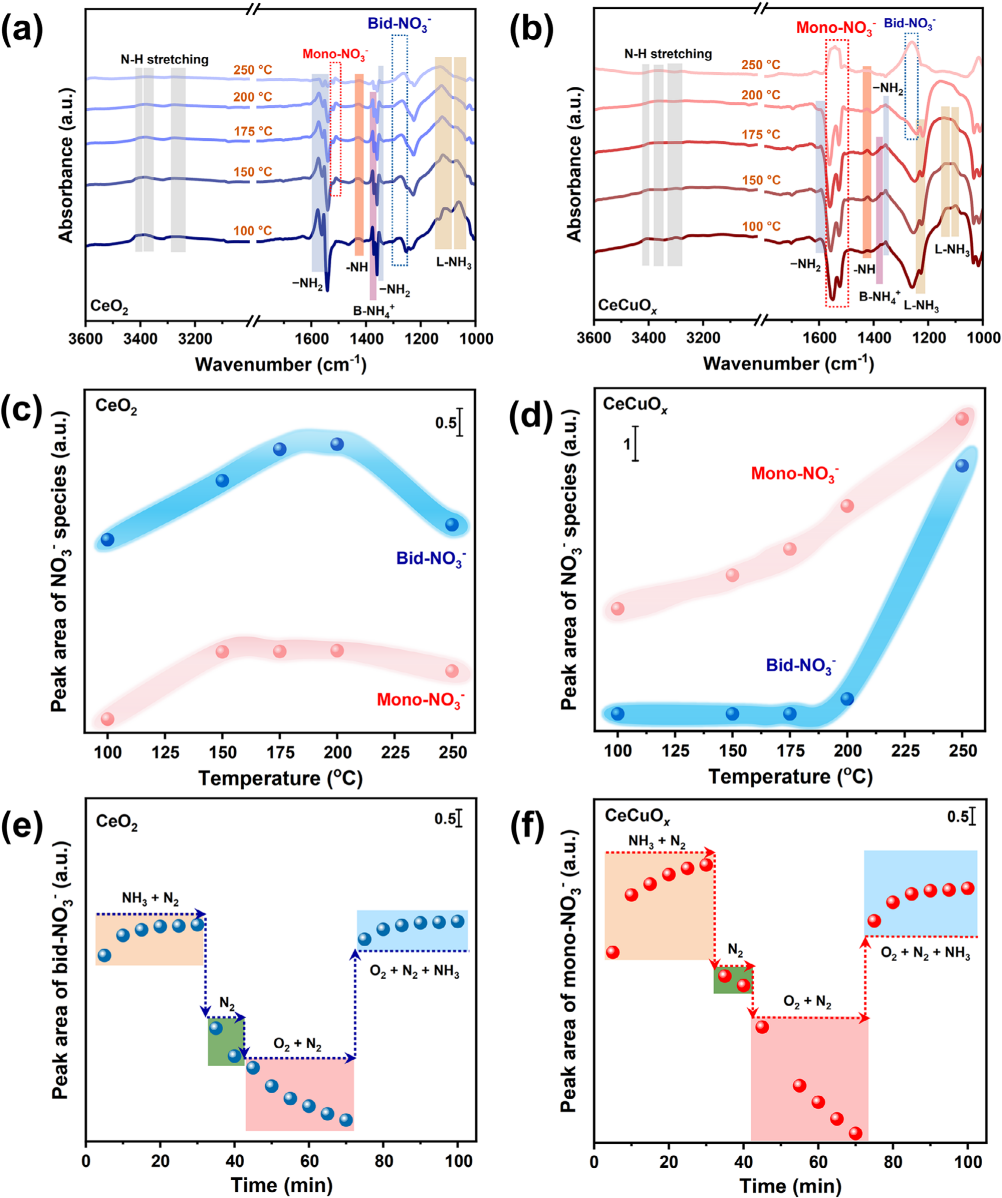
In situ DRIFTS and peak areas of the nitrate species of the a,c) CeO_2_ and b,d) CeCuO*_x_* catalysts, as a function of temperature, under NH_3_ oxidation reaction conditions. Peak areas associated with the e) bidentate nitrate species of the CeO_2_ catalyst and f) monodentate nitrate of the CeCuO*_x_* catalyst, obtained from in situ DRIFTS under atmosphere switching conditions at 200 °C.

In situ diffuse reflectance infrared Fourier transform spectroscopy (DRIFTS) was adopted to further investigate the NH_3_‐SCO reaction mechanism. **Figure** **6**a–d show the results of in situ DRIFTS over the CeO_2_ and CeCuO*_x_* catalysts in a stream of NH_3_ + O_2_ at 100–250 °C. As shown in Figure 6a, for CeO_2_, the bands at 1276, 1352, 1429, 1554, 1576, 3266, 3384, and 3415 cm^−1^ belonged to bidentate nitrate species (1276 cm^−1^),^[^ ^49^ ^]^ ‐NH_2_ stretching and bending vibrations (1352, 1554, and 1576 cm^−1^),^[^ ^50^, ^51^ ^]^ −NH stretching and bending vibrations (1429 cm^−1^),^[^ ^52^ ^]^ and N─H stretching vibrations (3266, 3384, and 3415 cm^−1^), respectively.^[^ ^53^ ^]^ NH_4_^+^ and NH_3_ species were adsorbed on the Brønsted acid sites (B‐NH_4_^+^, 1376 cm^−1^)^[^ ^50^ ^]^ and Lewis acid sites (L‐NH_3_; 1233, 1115, and 1062 cm^−1^),^[^ ^16^, ^54^ ^]^ respectively. Peaks associated with monodentate nitrate species (1525 and 1510 cm^−1^) on the CeO_2_ catalyst began to appear at 150 °C,^[^ ^55^, ^56^ ^]^ while those for bidentate nitrate species started appearing at 100 °C. To more intuitively assess the content changes for nitrate species, the peak areas associated with nitrate species were analyzed, and the results are shown in Figure 6c. As the temperature increased, nitrate species gradually accumulated, especially bidentate nitrate species, whose content was much higher than that of monodentate nitrate species, indicating that the main intermediate species on the CeO_2_ catalyst were bidentate nitrate species. With the addition of Cu, the main intermediate species switched from bidentate nitrate species to monodentate nitrate species (Figure 6b,d). To further explore the dynamic changes in the main intermediate species during NH_3_ oxidation, in situ DRIFT tests were performed under different atmospheric conditions. As shown in Figure S15a,b (Supporting Information), when NH_3_ was introduced into the reaction atmosphere, nitrate peaks were observed. Upon switching to a N_2_ atmosphere, the nitrate peaks could still be observed. However, with the addition of O_2_, the depletion of nitrate species occurred, indicating that the reaction of nitrate species with activated NH_3_ was the rate‐determining step. Figure 6e,f show the temporal evolution of bidentate nitrate species (main intermediate species) on the CeO_2_ catalyst, as well as monodentate nitrate species (main intermediate species) on the CeCuO*_x_* catalyst, respectively. It can be concluded that the nitrate species were consumed upon the introduction of O_2_, and the consumption rate for monodentate nitrate species on the CeCuO*_x_* catalyst was significantly higher than that for bidentate nitrate species on the CeO_2_ catalyst. Therefore, the CeCuO*_x_* catalyst exhibited higher NH_3_‐SCO catalytic performance compared to that of the CeO_2_ catalyst.

**Figure 7:**
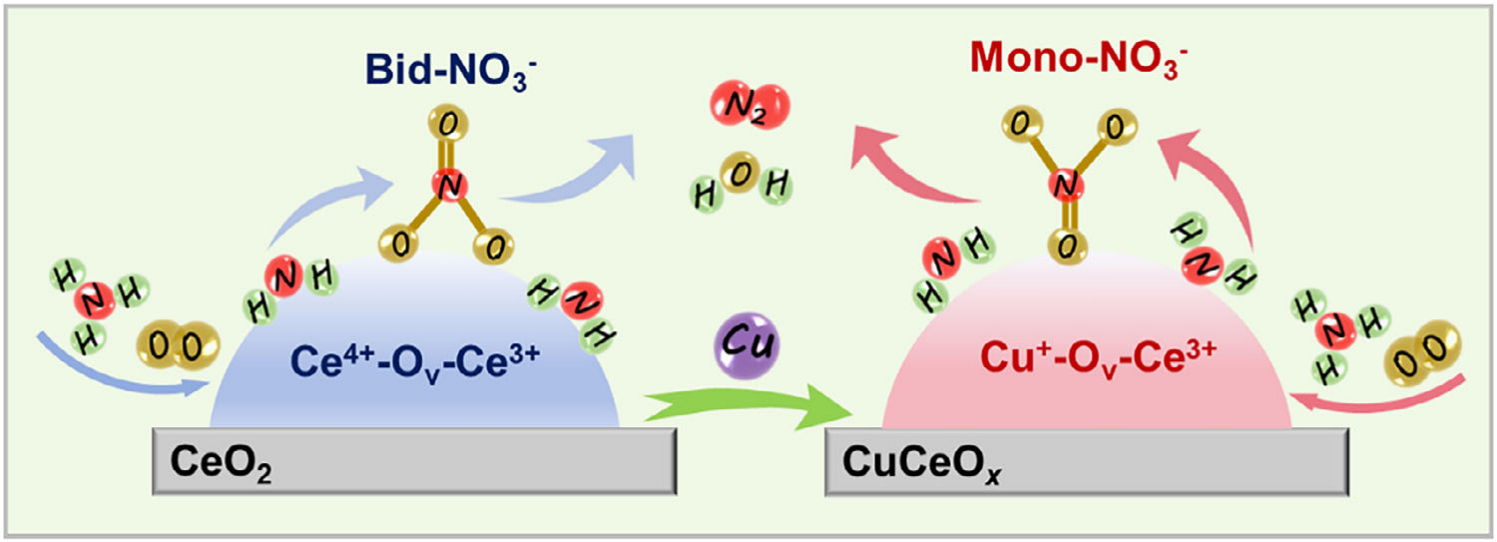
Mechanism diagram for NH_3_ oxidation over the CeO_2_ and CeCuO*_x_* catalysts.

A schematic diagram of the NH_3_‐SCO reaction mechanism for the CeO_2_ and CeCuO*_x_* catalysts is shown in **Figure** **7**. Doping Cu into CeO_2_ induced the formation of abundant Cu^+^‐O_v_‐Ce^3+^ sites, which were beneficial for the adsorption and activation of NH_3_ and O_2_. In addition, the addition of Cu switched the primary intermediate species of NH_3_‐SCO from bidentate nitrate species to monodentate nitrates, which reacted much more easily with activated NH_3_.

## 3 Conclusion

In summary, a remarkable CeCuO*_x_* catalyst for NH_3_‐SCO was developed through in situ hydrothermal synthesis. The CeCuO*_x_* catalyst exhibited superior NH_3_‐SCO activity, achieving 100% NH_3_ oxidation below 250 °C and N_2_ selectivity over 80%. Various characterization measurements and DFT calculations revealed that Cu doping into CeO_2_ induced the generation of abundant Cu^+^‐O_v_‐Ce^3+^ active sites, which could enhance the adsorption and activation of NH_3_ and O_2_. Additionally, doping with Cu induced a change in the main intermediate species for NH_3_‐SCO from bidentate nitrate species to monodentate nitrates, resulting in superior NH_3_‐SCO activity. These findings provide valuable insights into the design and construction of non‐noble‐metal NH_3_‐SCO catalysts with high activity and high N_2_ selectivity.

## 4 Experimental Section

### Catalyst Synthesis

Ce*_y_* Cu_1‐_ *_y_* O*_x_* catalysts (Ce/Cu molar ratio = 9.5/0.5, 9:1, 8.5/1.5) were prepared using a hydrothermal method. A specific amount of cerium(III) nitrate hexahydrate (Ce(NO_3_)_3_·6H_2_O, ≥99%; Tianjin Fuchen Chemical Reagents Factory) and copper(II) nitrate trihydrate (Cu(NO_3_)_2_·3H_2_O, ≥99%; Sinopharm Chemical Reagent Co., Ltd.) were dissolved in 30 mL of deionized H_2_O (denoted as solution A). 15 g of sodium hydroxide (NaOH, 96.0%; Sinopharm Chemical Reagent Co., Ltd.) were dissolved in 50 mL of deionized water (denoted as solution B). Solution A was added to solution B, and the resulting solution was stirred for 1 h. Subsequently, the mixed solution was transferred into a Teflon‐lined autoclave (100 mL) and then maintained in an oven at 100 °C for 12 h. Finally, the obtained precipitate was filtered, washed with deionized water and ethanol (C_2_H_5_OH, 99.9%; Sinopharm Chemical Reagent Co., Ltd.), dried, and calcined at 550 °C for 4 h to obtain Ce*_y_* Cu_1‐_ *_y_* O*_x_* catalysts. The catalyst with the best activity was named CeCuO*_x_*. The actual amounts of Ce and Cu in the CeCuO*_x_* catalyst were determined using inductively coupled plasma optical emission spectroscopy (ICP‐OES), and the results are shown in Table S2 (Supporting Information). For comparison, a pure CeO_2_ catalyst was synthesized using the same process but without the addition of Cu(NO_3_)_2_·3H_2_O, and a pure CuO (Aladdin) catalyst was calcined at 550 °C for 4 h.

### Catalyst Characterization

TEM and EDS images of the catalysts were obtained using a FEI Tecnai G^2^ F20 instrument. Moreover, N_2_ adsorption‐desorption experiments were conducted on a Micromeritics ASAP 2460 instrument cooled at −196 °C with liquid nitrogen. XRD patterns were collected by using a Bruker D8 Advance X‐ray diffractometer with Cu Kα radiation (λ = 1.5406 Å) at a scan step of 2°min^−1^ and in the 2θ range of 10–90°. XRD refinements of the samples were implemented using the Full Prof software. Factors such as zero‐correction parameters, scale, lattice parameters, the full width at half maximum, atomic positions, and temperature were considered free parameters. A pseudo‐Voigt function was used to simulate the experimental XRD patterns of the solid solutions. The actual amounts of Ce and Cu in the CeCuO*_x_* catalysts were determined via ICP‐OES. Raman tests were performed on a laser micro‐Raman spectrometer (Horiba HR Evolution) with an Ar laser beam at a wavelength of 532 nm and a power of 5 mW. XPS analysis was performed using a Thermo Escalab250Xi with a monochromatic Al Kα source operating at 120 W. The binding energies of all catalysts were calibrated using the C 1s peak at 284.8 eV. EPR spectra were obtained using a Bruker A300 spectrometer at −196 °C. The absorption edge and band‐gap energy (*E_g_*) were studied using UV–vis diffuse reflectance spectroscopy and recorded in the range of 200–800 nm (Shimadzu UV–3600i Plus), and BaSO_4_ was used as the internal standard. The optical *E_g_* was calculated from the plot between E = 1240/λ (nm), where λ is the wavelength corresponding to the adsorption edge, and (αhv)^2^(eV)^2^.

H_2_‐TPR, CO‐TPR, O_2_‐TPD, and in situ O_2_‐pulse experiments were performed using a Micromeritics AutoChem II 2930 instrument equipped with a thermal conductivity detector (TCD). In each experiment, catalysts (100 mg) were placed into a U‐type quartz tube. Before analysis, all fresh catalysts underwent in situ pretreatment in a flow of 5 vol.% O_2_/He (30 mL min^−1^) at 300 °C for 1 h, after which they were cooled down to room temperature. Subsequently, purging with He for 30 min at room temperature was performed to remove species adsorbed on the catalyst surfaces. For the H_2_‐TPR test, samples were heated from room temperature to 1000 °C at 10 °C min^−1^ under 10 vol.% H_2_/Ar (30 mL min^−1^). CO‐TPR experiments were performed in a manner similar to that used for the H_2_‐TPR experiment, but using 5 vol.% CO/He. For the O_2_‐TPD test, samples were first exposed to a 5 vol.% O_2_/He atmosphere (30 mL min−) for 1 h, and then the temperature was raised from room temperature to 1000 °C in He flow at a rate of 10 °C min^−1^. The outlet exhaust was detected using a mass spectrometer (MS, MKS Cirrus 3) equipped with a quadrupole detector. For the in situ O_2_‐pulse test, the catalyst was treated under a He atmosphere at 300 °C for 30 min and then cooled to room temperature in the He atmosphere. The sample was then heated from room temperature to 50, 100, 150, 175, 200, 225, and 250 °C, respectively, and 0.4 vol.% CO/He was introduced for purging until the baseline was stable at each reaction temperature. Subsequently, 5 vol.% O_2_/He pulses (0.5 mL) were injected at each reaction temperature. The outlet exhaust was detected using a MS equipped with a quadrupole detector.

NH_3_‐TPD experiments were performed using an Antaris IGS (Thermo Fisher). The catalyst was pretreated with 10 vol.% O_2_/N_2_ (200 mL min^−1^) at 200 °C for 1 h and then cooled down to 30 °C. Thereafter, the catalyst was exposed to NH_3_/N_2_ (500 ppm) for 1 h, followed by N_2_ purging for 30 min. Finally, the reaction temperature was increased from 30 to 300 °C at a rate of 5 °C min^−1^.

The NH_3_‐SCO reaction mechanism was explored via in situ DRIFTS using a Thermo Fisher iS50 FTIR instrument equipped with an in situ diffuse reflectance chamber (Harrick) and a high‐sensitivity MCT/A detector. All spectra were collected at a resolution of 4 cm^−1^ and using an accumulation of 100 scans at 4000–650 cm^−1^. Before the test, samples were pretreated at 300 °C in a flow of air (100 mL min^−1^) for 30 min, and cooled down to 100/150/175/200/250 °C, and then purged with N_2_ to obtain the background spectra. Subsequently, a mixture (500 ppm of NH_3_ and 10 vol.% O_2_, balanced with N_2_) was added to the reaction atmosphere for 30 min at each temperature. Moreover, the NH_3_ oxidation reaction mechanism was investigated by switching the atmosphere. Samples were pretreated at 300 °C in a flow of air (100 mL min^−1^) for 30 min, and cooled down to 200 °C, and purged with N_2_ to obtain the background spectra. NH_3_ was first pre‐adsorbed for 30 min, followed by purging with N_2_ for 10 min; thereafter, 10 vol.% O_2_ was then introduced for 30 min, and finally the atmosphere was switched to NH_3_ (500 ppm), 10 vol.% O_2_, and N_2_ for 30 min.

### Catalytic Performance Evaluation

NH_3_ oxidation activity tests were conducted in a continuous‐flow fixed‐bed quartz reactor with an inner diameter of 5 mm, and catalysts (100 mg, 40–60 mesh) were used for the tests. The reaction gas mixture consisted of NH_3_ (500 ppm) and O_2_ (10 vol.%), balanced with N_2_ at a total flow rate of 200 mL min^−1^. Long‐term stability tests were then performed under the same reaction gas conditions, maintaining a specific temperature of 225/300 °C. The outlet gases were analyzed using an Antaris IGS (Thermo Fisher) equipped with a heated and low‐volume multiple‐path gas cell (2 m). The NH_3_ conversion, N_2_ selectivity, NO selectivity, NO_2_ selectivity, N_2_O selectivity, and reaction rate (*r*) were calculated as follows:

(1) $$NH_{3}\;\mathrm{conversion}=\left(1-\frac{{\left[NH_{3}\right]}_{\mathrm{out}}}{{\left[NH_{3}\right]}_{\mathrm{in}}}\right)\times 100\%$$

(2) $$\begin{array}{ccc} N_{2}\;\mathrm{selectivity} & = & \left(\frac{{\left[NH_{3}\right]}_{\mathrm{in}}-{\left[NO_{2}\right]}_{\mathrm{out}}-{\left[\mathrm{NO}\right]}_{\mathrm{out}}-2{\left[N_{2}O\right]}_{\mathrm{out}}}{{\left[NH_{3}\right]}_{\mathrm{in}}}\right)\\ & & \times 100\% \end{array}$$

(3) $$\mathrm{NO}\;\mathrm{selectivity}=\left(\frac{{\left[\mathrm{NO}\right]}_{\mathrm{out}}}{{\left[NH_{3}\right]}_{\mathrm{in}}-{\left[NH_{3}\right]}_{\mathrm{out}}}\right)\times 100\%$$

(4) $$NO_{2}\;\mathrm{selectivity}=\left(\frac{{\left[NO_{2}\right]}_{\mathrm{out}}}{{\left[NH_{3}\right]}_{\mathrm{in}}-{\left[NH_{3}\right]}_{\mathrm{out}}}\right)\times 100\%$$

(5) $$N_{2}O\;\mathrm{selectivity}=\left(\frac{2{\left[N_{2}O\right]}_{\mathrm{out}}}{{\left[NH_{3}\right]}_{\mathrm{in}}-{\left[NH_{3}\right]}_{\mathrm{out}}}\right)\times 100\%$$

(6) $$r_{NH_{3}}=\frac{F_{NH_{3}}X_{NH_{3}}}{m}\left(\mathrm{mol}/g/s\right)$$

where *m* (g) is the mass of the catalyst used for the measurement, *F_NH3_* (mol/s) is the flow rate of NH_3_, and *X_NH3_* is the conversion of NH_3_.

### Kinetic Measurements

The device used for the kinetic measurement, the composition of the reaction gas mixture, and the total flow rate were consistent with the catalytic‐performance evaluation. The WHSV of the CeO_2_, CuO, and CeCuO*_x_* catalysts were 400 000, 600 000, and 1 200 000 mL g^−1^ h^−1^, respectively. To minimize any potential influence of reactant diffusion, the activation energies for the NH_3_ oxidation reaction were calculated based on reaction rates measured at low NH_3_ conversion (less than 20%), using the Arrhenius Equation (7):

(7) $$\ln r=-\frac{E_{a}}{RT}+\ln A$$

where *E_a_*, *T*, and *A* represent the apparent activation energy (kJ/mol), the reaction temperature (K), and the pre‐exponential factor (s^−1^), respectively.

## Conflict of Interest

The authors declare no conflict of interest.

## Supporting information



Supporting Information

## Data Availability

The data that support the findings of this study are available from the corresponding author upon reasonable request.
