# Supplementary material for: In Situ Construction of Cu+‐Ov‐Ce3+ Sites on CeO2 for Efficient NH3 Oxidation
Source: Adv Sci (Weinh). 2025 Sep 18;12(47):e11023. doi: 10.1002/advs.202511023 (PMC12713011; doi:10.1002/advs.202511023)
Supplement: Supplementary file 1 — Supporting Information [file ADVS-12-e11023-s001.docx]

Supporting Information

**In Situ Construction of Cu^+^-O_v_-Ce^3+^ Sites on CeO_2_ for Efficient NH_3_ Oxidation**

C. Duan, M. Wang, Y. Zhang, Z. Lian, W. Shan

State Key Laboratory of Advanced Environmental Technology, Institute of Urban Environment, Chinese Academy of Sciences, Xiamen 361021, China
E-mail: mwang@iue.ac.cn (M. Wang), yzhang3@iue.ac.cn (Y. Zhang), wpshan@iue.ac.cn (W. Shan)

C. Duan, M. Wang, Y. Zhang, W. Shan
Zhejiang Key Laboratory of Pollution Control for Port-Petrochemical Industry, Ningbo Urban Environment Observation and Research Station, Institute of Urban Environment, Chinese Academy of Sciences, Ningbo 315800, China

E-mail: mwang@iue.ac.cn (M. Wang), yzhang3@iue.ac.cn (Y. Zhang), wpshan@iue.ac.cn (W. Shan)

C. Duan, Y. Zhang, W. Shan

University of Chinese Academy of Sciences, Beijing 100049, China

E-mail: yzhang3@iue.ac.cn (Y. Zhang), wpshan@iue.ac.cn (W. Shan)

**DFT Calculations**

The spin-polarized DFT calculations were carried out using the projector augmented-wave (PAW) method within the Vienna ab-initio simulation package (VASP) together with the Perdew-Burke-Ernzerhof (PBE) functional.^[1-3]^ The DFT+U method was employed with an effective U_eff_ value of 5.0 eV for Ce 4f.^[4]^ The CeO_2_ (111) was created as the slab model with three O-Ce-O layers, a 231 supercell, and a vacuum layer of 15 Å. For the model of the CeCuO*_x_* catalyst, four Ce atoms were replaced by Cu atoms randomly, and eight additional H atoms were introduced to counteract the charged state induced by the substitutional doping of Cu. The 111 K-points, cutoff energy of 450 eV, and Grimme D3 dispersion correction method were used for structure relaxation and single-point energy calculations, with the convergence criteria of 1 × 10^-5^ eV and 0.02 eV Å^-1^ for energy and force, respectively.^[5]^ The adsorption energies of NH_3_ and O_2_ were calculated using the following equations:

*E_ad,NH3_* = *E_substrate+NH3_* - *E_substrate_* - *E_NH3_*

*E_ad,O2_* = *E_substrate+O2_* - *E_substrate_* - *E_O2_*

where *E_ad_* is the adsorption energy, *E_substrate+O2/NH3_* is the total energy of O_2_ or NH_3_ adsorbed on the substrate, *E_O2/NH3_* is the total energy of O_2_ or NH_3_, and *E_substrate_* is the total energy of the substrate.

The defect formation energy (*E_def_*) was defined as:

*E_def_* = *E_Ov_* + *n_o_μ_o_* - *E_slab_*

where *E_Ov_* and *E_slab_* are the total energies of the slab model with and without an oxygen vacancy, respectively; *n_o_* and *μ_o_* represent the number and chemical potential of the removed O atom, respectively, where *μ_o_* is approximately half of the total energy of an O_2_ molecule in a vacuum.


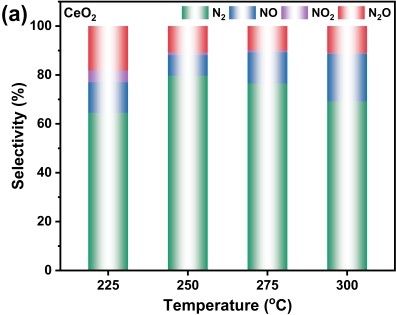

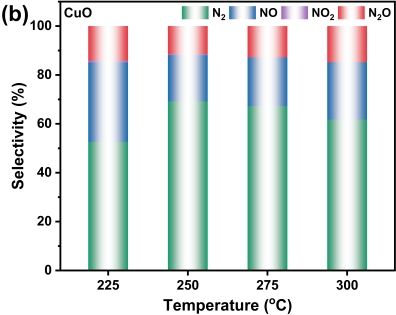

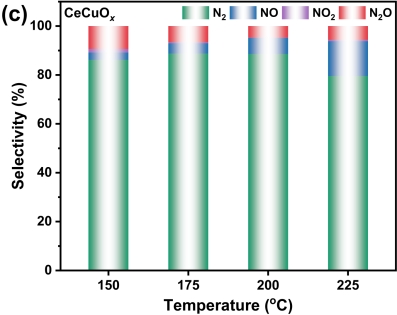


**Figure S1.** Selectivity of (a) CeO_2_, (b) CuO, and (c) CeCuO*_x_* catalysts. Reaction conditions: 500 ppm of NH_3_ and 10 vol.% O_2_ in N_2_ balance; WHSV = 120000 mL/g/h.


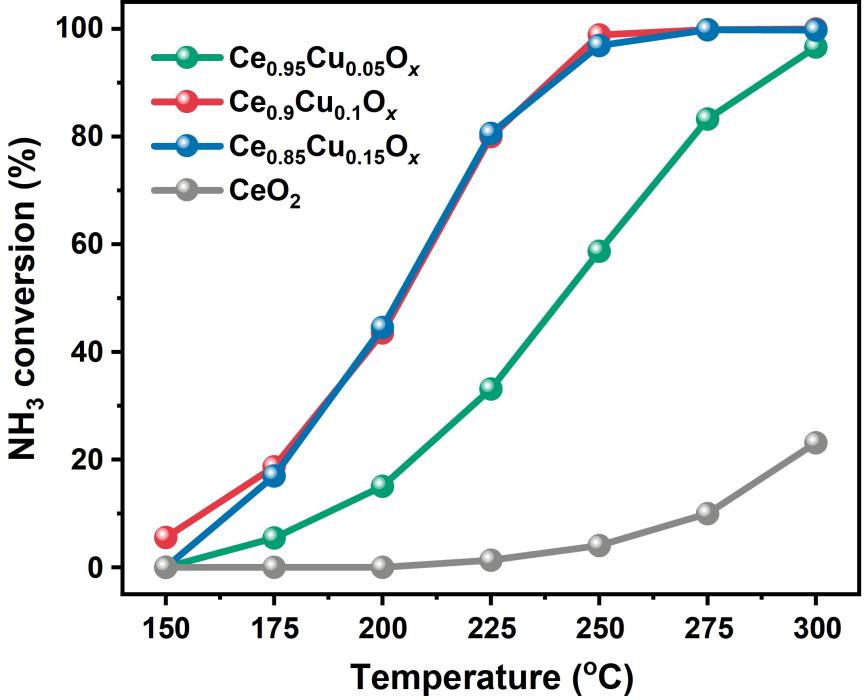


**Figure S2.** NH_3_ conversion of CeCuO*_x_* catalysts with different Ce/Cu ratios.


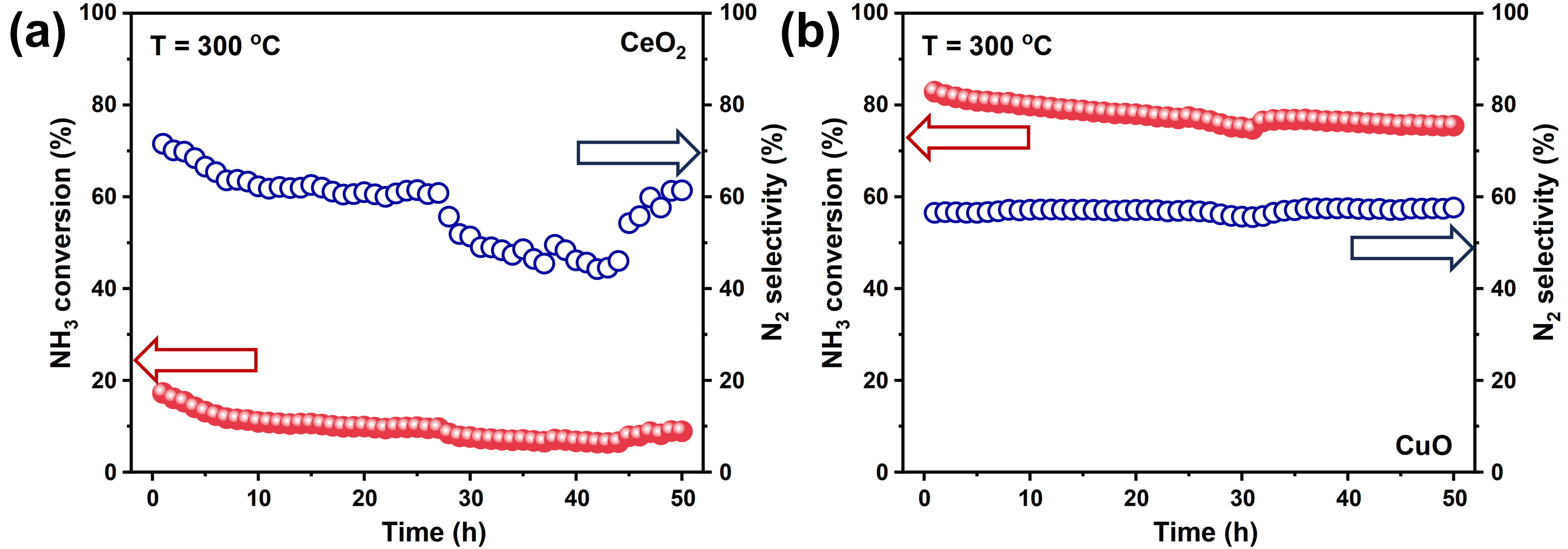


**Figure S3.** NH_3_ conversion and N_2_ selectivity of (a) CeO_2_ and (b) CuO catalysts during a long-term stability test at 300 °C.


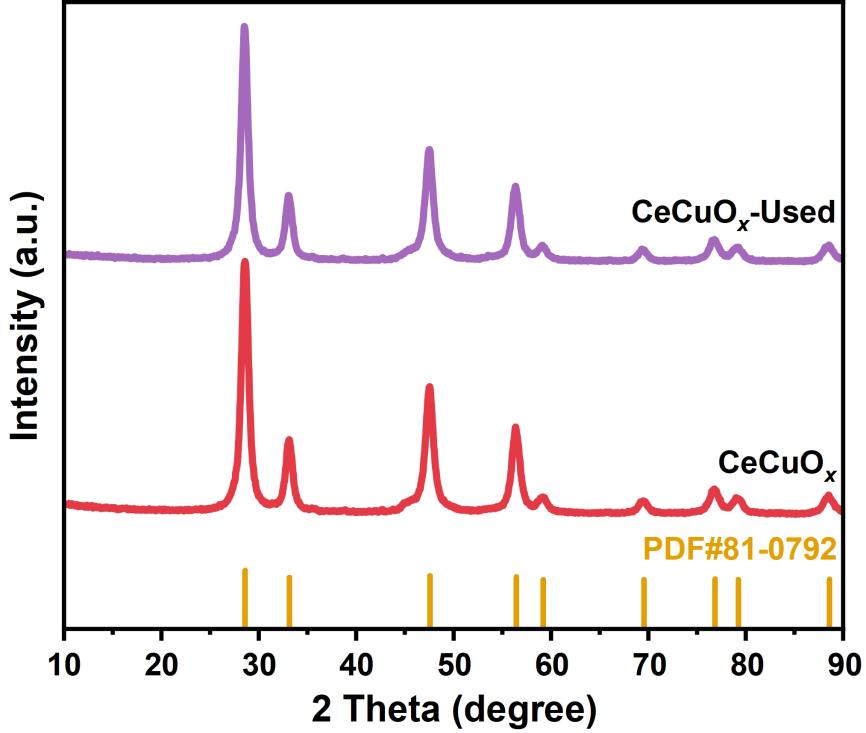


**Figure S4.** The XRD patterns of fresh and spent CeCuO*_x_* catalyst.


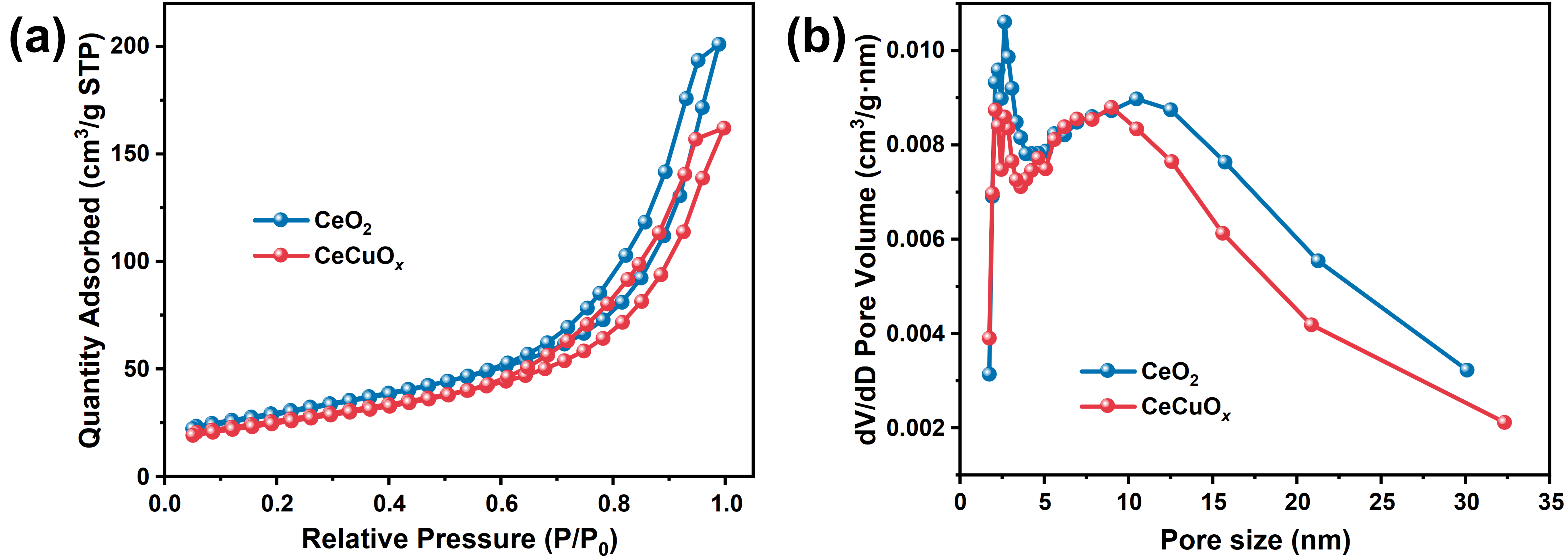


**Figure S5.** (a) N_2_ adsorption-desorption isotherms and (b) pore size distributions of CeO_2_ and CeCuO*_x_* catalysts.


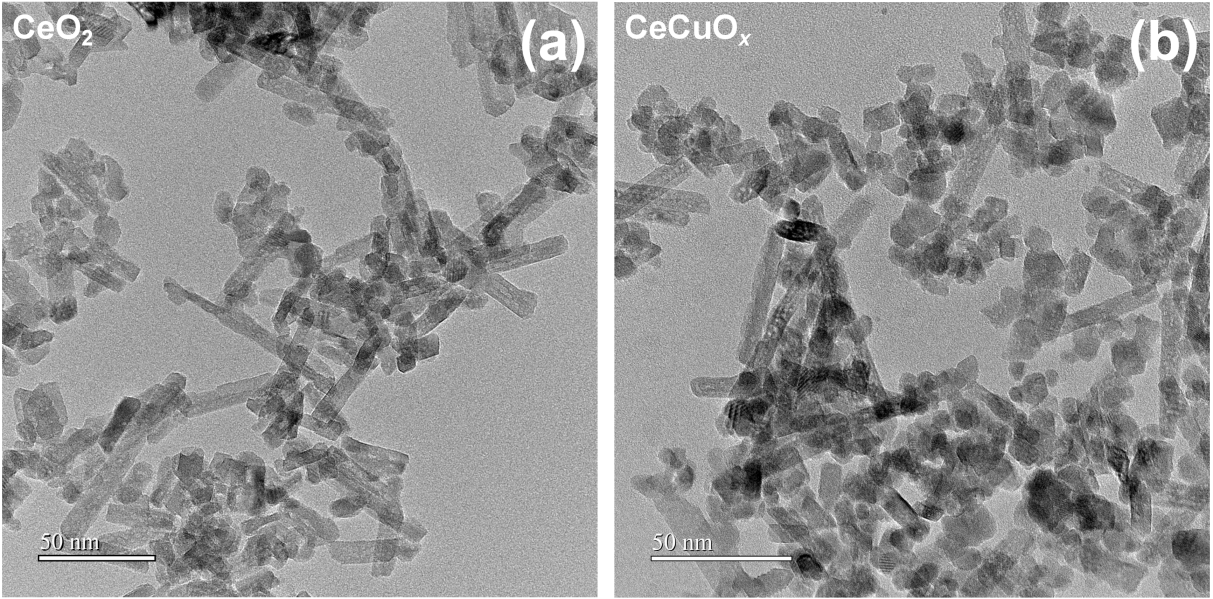


**Figure S6.** TEM images of (a) CeO_2_ and (b) CeCuO*_x_* catalysts.


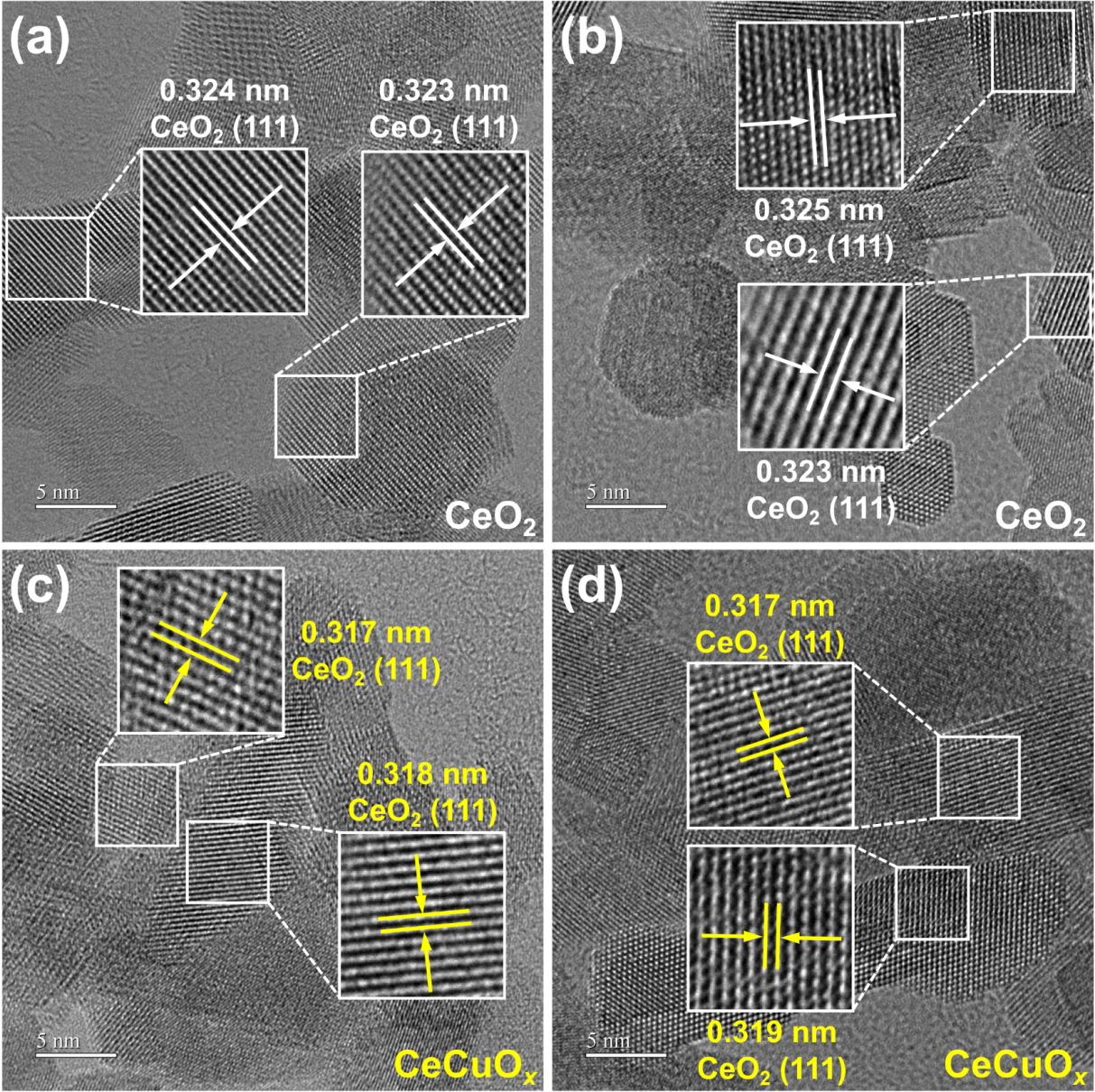


**Figure S7.** HRTEM images of (a, b) CeO_2_ and (c, d) CeCuO*_x_* catalysts.


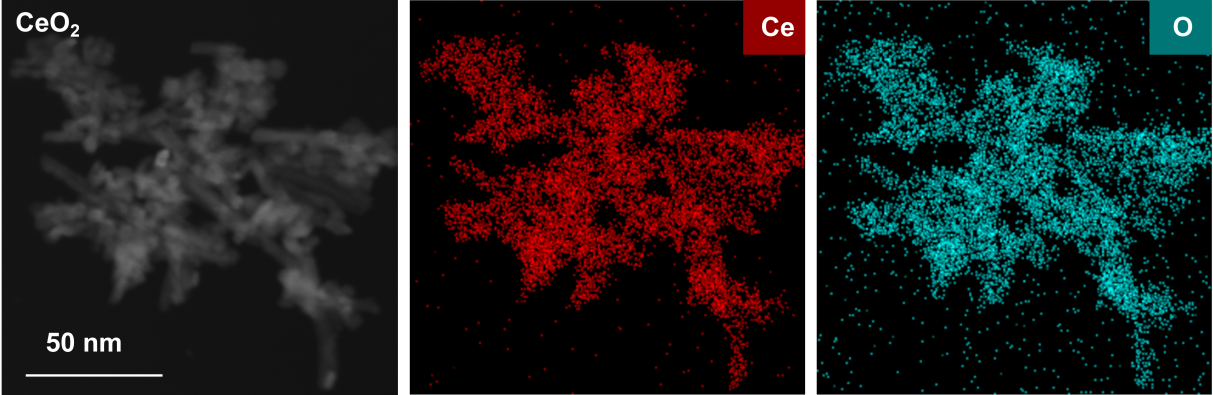


**Figure S8.** STEM-EDS mapping images of the CeO_2_ catalyst.


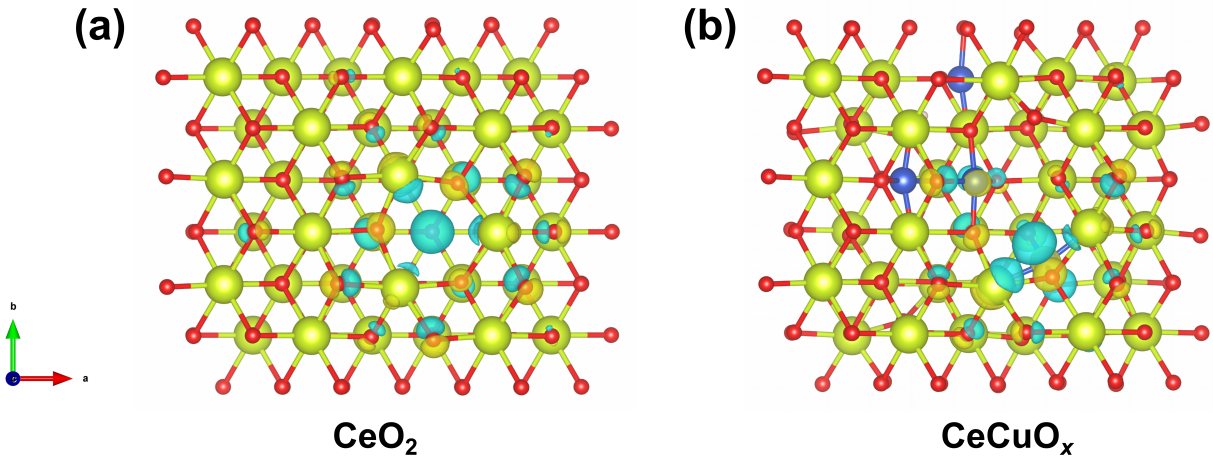


**Figure S9.** Charge density difference of (a) CeO_2_ and (b) CeCuO*_x_* catalysts. Yellow and cyan regions represent electron accumulation and electron depletion, respectively. Light green, Ce atoms; red, O atoms; blue, Cu atoms; the value of the iso-surface is 0.1 e/bohr^3^.


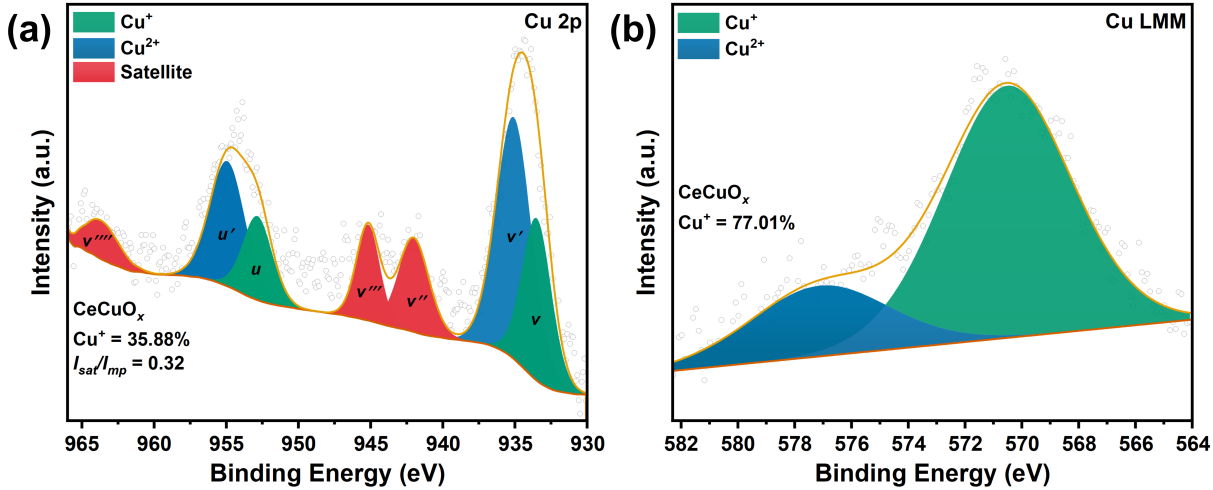


**Figure S10.** (a) Cu 2p and (b) Cu LMM Auger spectra of the CeCuO*_x_* catalyst.


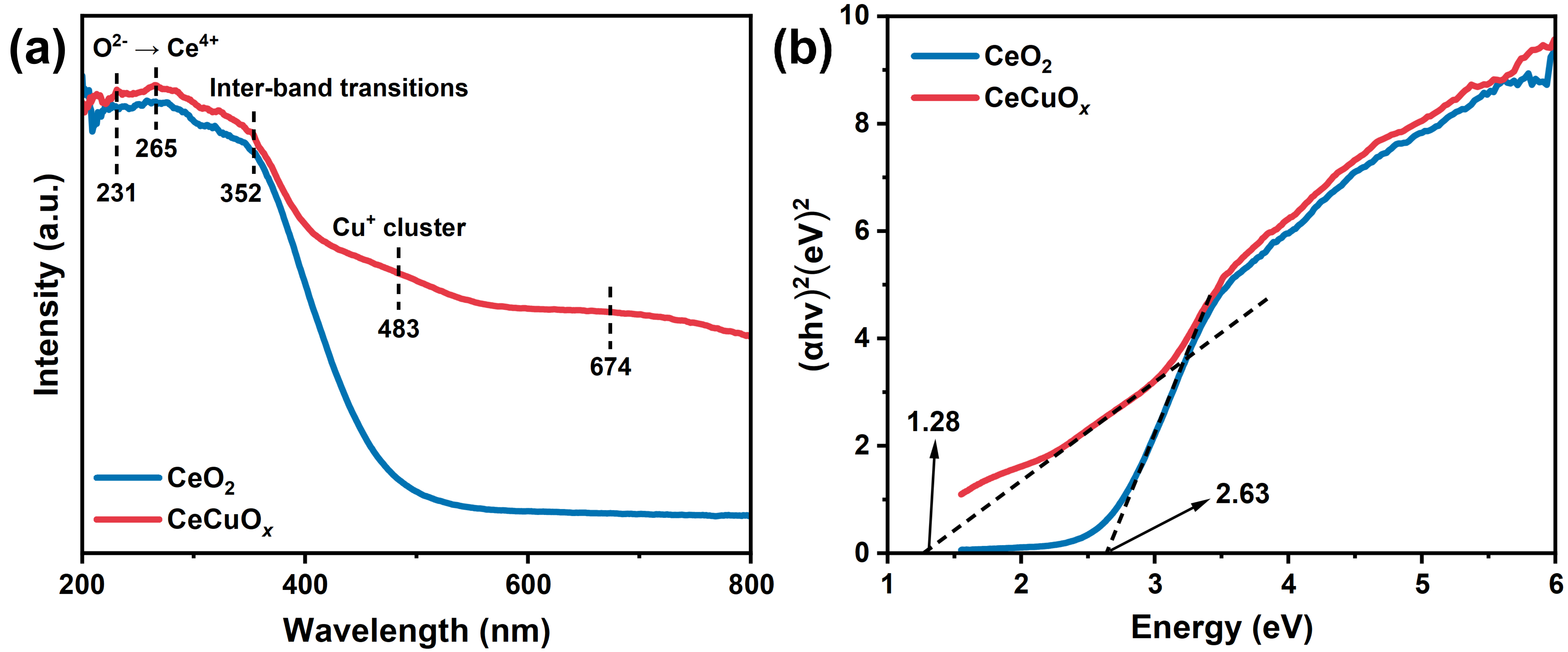


**Figure S11.** (a) UV-vis diffuse reflectance spectra in the range of 200-800 nm and (b) the plot of (αhv)^2^(eV)^2^ versus for the *E_g_* of CeO_2_ and CeCuO*_x_* catalyst.


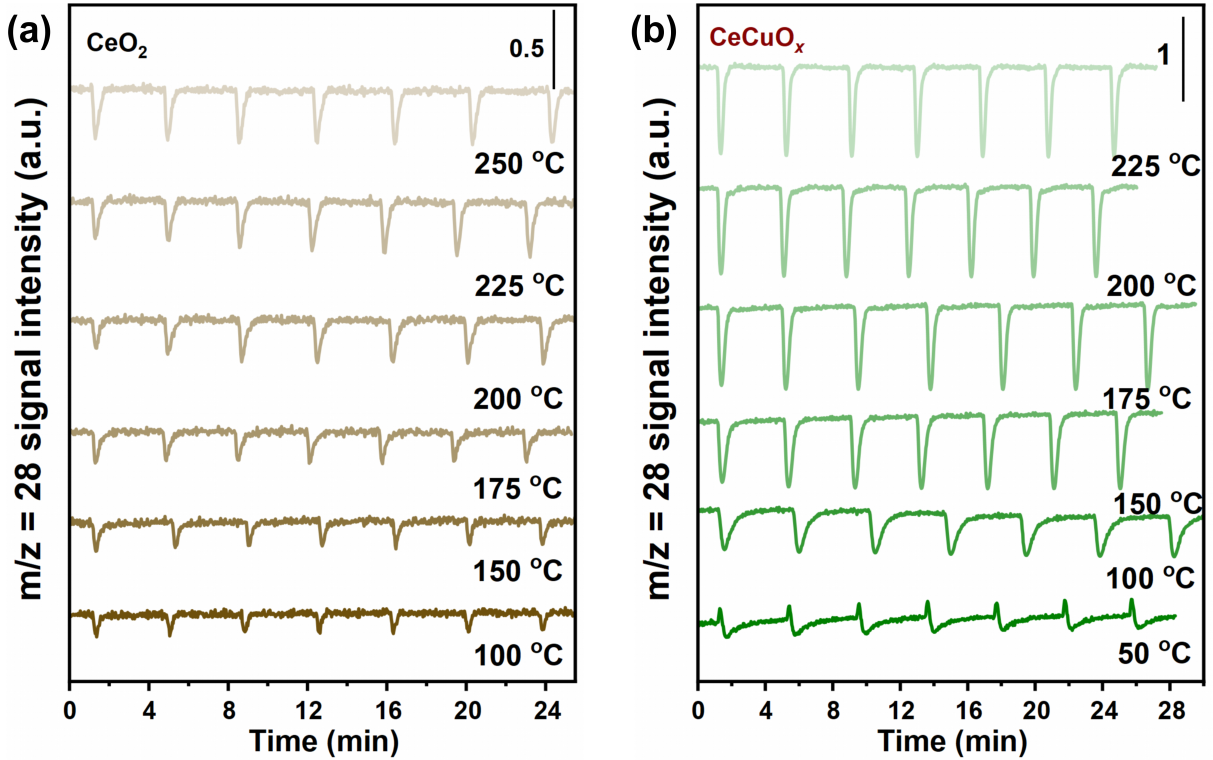


**Figure S12.** *In situ* O_2_-Pulse spectra of CO signals for (a) CeO_2_ and (b) CeCuO*_x_* catalysts.


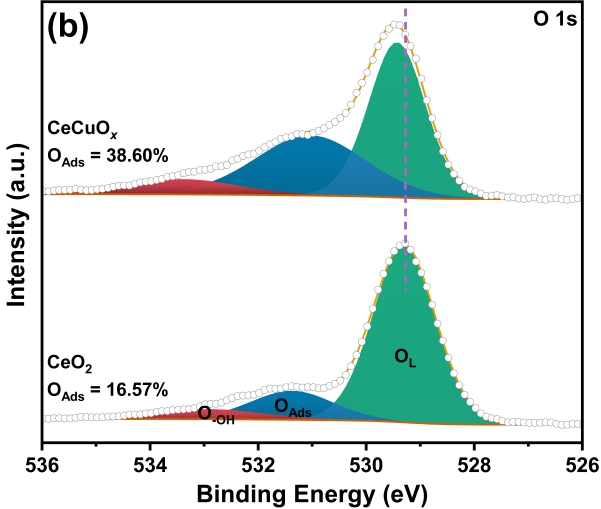

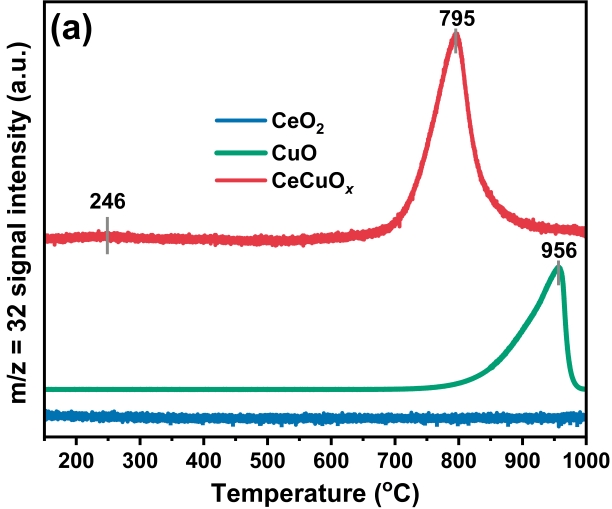


**Figure S13.** (a) O_2_-TPD profiles of the as-synthesized catalysts and (b) O 1s XPS spectra of CeO_2_ and CeCuO*_x_* catalysts.


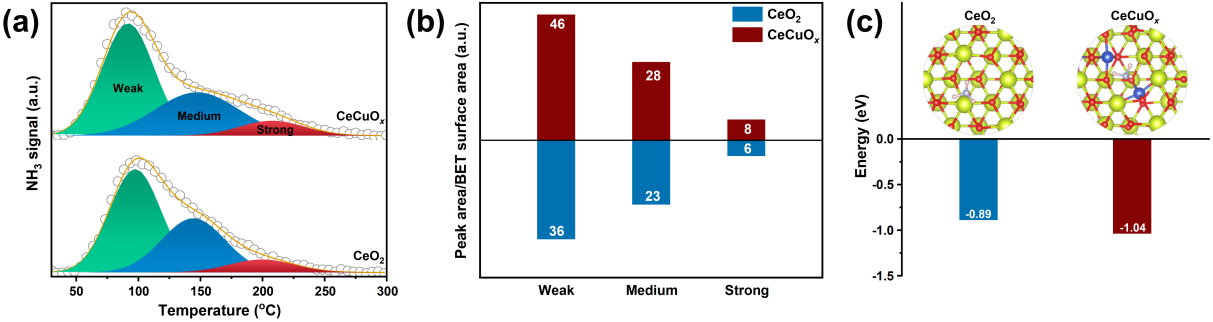


**Figure S14.** (a) NH_3_-TPD profiles and (b) peak area of different acid sites/BET surface area of CeO_2_ and CeCuO*_x_* catalysts. (c) NH_3_ adsorption energy of CeO_2_ and CeCuO*_x_* catalysts.


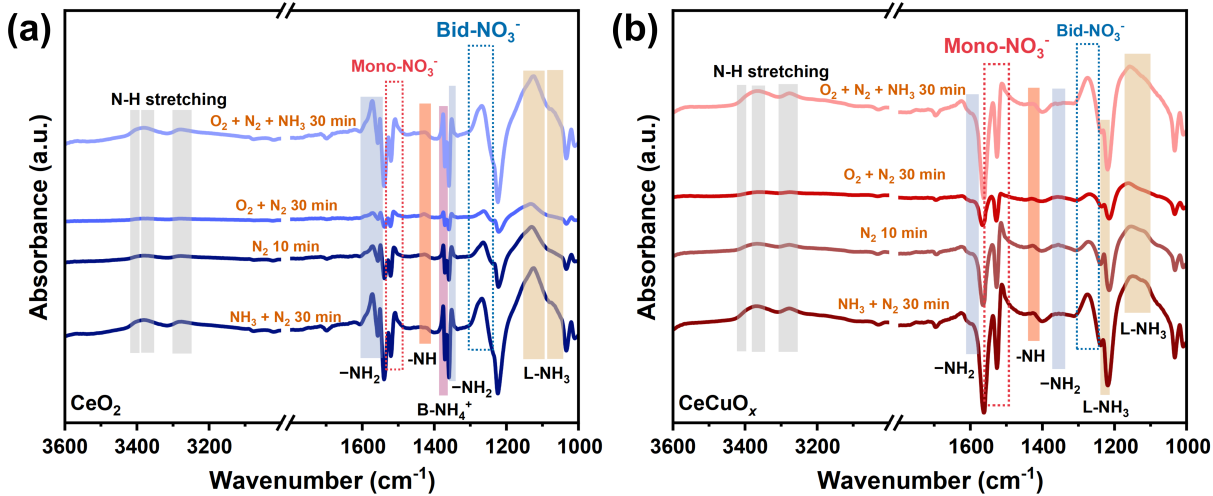


**Figure S15.** In situ DRIFTS results of (a) CeO_2_ and (b) CeCuO*_x_* catalysts under different reaction atmosphere conditions at 200 ℃.

**Table S1.** BET surface area, pore volume, and average pore size of CeO_2_ and CeCuO*_x_* catalysts.

| **Catalysts** | **BET surface area (m^2^/g)^a)^** | **Pore volume (cm^3^/g)^b)^** | **Average pore size (nm)^c)^** |
| --- | --- | --- | --- |
| CeO_2_ | 105.9 | 0.311 | 11.74 |
| CeCuO*_x_* | 92.2 | 0.248 | 10.76 |

^a)^ Determined by BET method; ^b)^ Determined by BET method; ^c)^ Determined by BJH method according to the N_2_ desorption isotherm.

**Table S2.** The actual amounts of Ce and Cu in the CeCuO*_x_* catalyst by ICP-OES.

| **Catalyst** | **Ce (wt.%)** | **Cu (wt.%)** |
| --- | --- | --- |
| CeCuO*_x_* | 74.15 | 3.50 |

**Reference**

1. Kresse, G.; Joubert, D. From ultrasoft pseudopotentials to the projector augmented-wave method. *Phys. Rev. B* **1999**, *59*, 1758-1775.
2. Kresse, G.; Furthmuller, J. Efficient iterative schemes for ab initio total-energy calculations using a plane-wave basis set. *Phys. Rev. B* **1996**, *54*, 11169-11186.
3. Perdew, J. P.; Burke, K.; Ernzerhof, M. Generalized gradient approximation made simple. *Phys. Rev. Lett.* **1996**, *77*, 3865-3868.
4. Zheng, K.; Li, Y. F.; Liu, B.; Jiang, F.; Xu, Y. B.; Liu, X. H. Ti-doped CeO_2_ stabilized single-Atom rhodium catalyst for selective and stable CO_2_ hydrogenation to ethanol. *Angew. Chem. Int. Ed.* **2022**, *61*, e202210991.
5. Grimme, S.; Ehrlich, S.; Goerigk, L. Effect of the damping function in dispersion corrected density functional theory. *J. Comput. Chem.* **2011**, *32*, 1456-1465.
